# Are Leg Muscle, Tendon and Functional Characteristics Associated with Medial Tibial Stress Syndrome? A Systematic Review

**DOI:** 10.1186/s40798-021-00362-2

**Published:** 2021-10-09

**Authors:** Joshua P. M. Mattock, Julie R. Steele, Karen J. Mickle

**Affiliations:** 1 Biomechanics Research Laboratory, Faculty of Science, Medicine & Health, University of Wollongong, Northfields Avenue, Wollongong, NSW 2522 Australia; 2 School of Allied Health, Human Services and Sport, La Trobe University, Melbourne, VIC Australia

**Keywords:** Shin splints, Injury prevention, Injury rehabilitation, Leg girth

## Abstract

**Background:**

Medial tibial stress syndrome (MTSS) is a common overuse injury that lacks effective evidence-based treatment options. Reduced leg girth has been associated with MTSS development because it is hypothesised to impair the ability of the leg to modulate tibial loading generated during foot–ground contact. Measuring total leg girth, however, does not provide specific information about the structural composition or functional capacity of individual leg muscles. Consequently, uncertainty remains as to which specific muscles are compromised and contribute to MTSS development. Therefore, this paper aimed to systematically review the body of literature pertaining to how the structure and function of the leg muscles are thought to be associated with MTSS injury.

**Methods:**

The review was conducted following the Preferred Reporting Items for Systematic Reviews and Meta-Analysis Protocols (PRISMA-P). Medline, PubMed, SCOPUS, SPORTDiscus with Full-texts and Web of Science were searched until March 2021 to identify articles in which lower limb muscle structural or functional variables associated with MTSS injury were investigated.

**Results:**

Seventeen studies, which were predominately case–control in design and captured data from 332 individuals with MTSS symptoms and 694 control participants, were deemed appropriate for review. The average Downs and Black Quality Assessment score was 71.7 ± 16.4%, with these articles focussing on leg girth, tendon abnormalities, muscle strength and endurance, shear modulus and neuromuscular control. Of the risk factors assessed in the 17 studies, decreased lean leg girth and higher peak soleus muscle activity during propulsion were most strongly correlated with MTSS development. Individuals with MTSS also displayed deficits in ankle plantar flexor endurance, greater isokinetic concentric eversion strength, increased muscle shear modulus and altered neuromuscular recruitment strategies compared to asymptomatic controls.

**Conclusions:**

Future prospective studies are required to confirm whether decreased lean leg girth and higher peak soleus muscle activity during propulsion are associated with MTSS development and to elucidate whether these structural and functional differences in the leg muscles between MTSS symptomatic and asymptomatic controls are a cause or effect of MTSS.

**Supplementary Information:**

The online version contains supplementary material available at 10.1186/s40798-021-00362-2.

## Key Points

- Decreased lean leg girth is a likely risk factor associated with developing MTSS, although this reduction in muscle girth is not related to the capacity of the leg muscles to produce maximal force.
- Higher peak soleus muscle activity during propulsion is likely associated with MTSS development.
- The small number of prospective studies deemed appropriate to include in this review resulted in large knowledge gaps as to how leg muscle structure and function are associated with MTSS. Therefore, future prospective studies are necessary to assess leg muscle structural and functional characteristics in populations at risk of MTSS development.

## Background

Running is a popular form of physical activity primarily driven by the physical health benefits associated with exercise and the relative ease of accessibility to running [1]. Although the health benefits associated with running are widely acknowledged [2], runners of all levels and disciplines experience overuse injuries, with those training for longer distances at greater risk of injury [3]. Overuse injuries can be problematic for runners because these injuries have the potential to disrupt active lifestyles and negatively influence the positive physiological adaptations gained from running [4].

One overuse injury afflicting both novice and experienced runners is medial tibial stress syndrome (MTSS), more commonly referred to using the outdated term, shin splints. With an incidence rate of between 4 and 35% [5–7], MTSS predominately affects individuals participating in activities that impose repetitive loading upon the lower limb, such as running [8]. Pain is diffuse, covering an area of at least 5 cm at the middle to distal third of the posteromedial tibial border [5]. MTSS is a separate pathology from a stress fracture, chronic exertional compartment syndrome and neuropathies affecting the lower limb [6].

To better inform MTSS treatment protocols, numerous research studies have been conducted to identify risk factors for developing the injury. The authors of a systematic review and meta-analysis reported that the risk factors for developing MTSS included female sex, previous history of MTSS, fewer years running experience, orthotic use, increased body mass index (BMI), increased navicular drop and greater hip external rotation range of motion in males [8]. Difficulty exists, however, in basing MTSS treatment protocols on these current risk factors because many of them cannot be easily modified. Furthermore, there is no high-quality evidence for the effect of any current intervention in managing MTSS based on these risk factors [9].

One risk factor proposed to contribute to developing MTSS, which could be modified, but is yet to be extensively explored, is reduced leg girth [10]. Reduced leg musculature is thought to impair the ability of the leg to modulate tibial loading caused by the ground reaction forces generated at foot–ground contact during the stance phase of running, resulting in increased tibial loading placing individuals at risk of developing MTSS [10]. Although leg girth provides a gross measure of muscular bulk, it does not provide detail as to the structural composition or functional capacity of the leg muscles. Therefore, we do not know which specific leg muscles might be compromised in individuals with MTSS or how this might impact lower limb function. However, leg muscle structure and function could be targeted and relatively easily modified with appropriate interventions to modulate tibial loading and reduce MTSS incidence.

Several research teams have assessed leg muscle structural and functional characteristics in individuals with and without MTSS [10–24]. To date, however, no publication could be located in which the outcomes of these studies were systematically reviewed or how changes to leg muscle structure and function might predispose individuals to MTSS injury. The long recovery period from MTSS symptoms [25] combined with the high MTSS incidence rate highlight that further exploration of modifiable risk factors for MTSS is needed to develop better treatment protocols. Therefore, this paper aimed to systematically review the body of literature pertaining to how the structure and function of the leg muscles were associated with MTSS injury. Our secondary aim was to develop recommendations to direct future research studies to fill knowledge gaps related to leg structure and function with the ultimate goal of better informing MTSS treatment protocols.

## Methods

### Literature Search Strategy

To conform to best practice guidelines for systematic literature reviews, we conducted this review following the Preferred Reporting Items for Systematic Reviews and Meta-Analysis Protocols (PRISMA-P) (see Additional file 1: Appendix 1 *Prisma-P checklist*). The review protocol was registered on the PROSPERO international prospective register for systematic reviews website (https://www.crd.york.ac.uk/prospero) (PROSPERO 2020 CRD42020154523). The question for this systematic literature review was 'Do individuals who develop and are afflicted with MTSS display differences in lower leg muscle structure or function compared to active asymptomatic individuals?'.

**Fig. 1:**
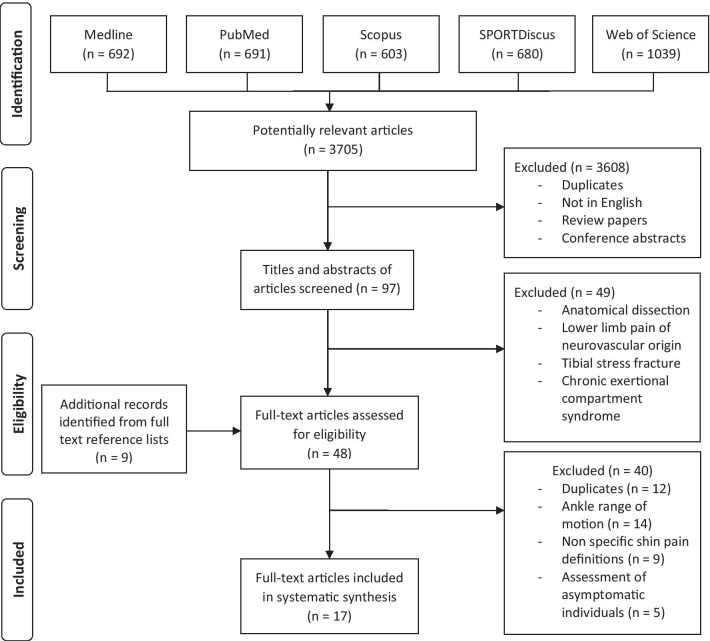
Systematic review flow diagram

A systematic literature search was completed in March 2021, and relevant articles in which the authors had investigated leg muscle structure or function associated with MTSS injury were identified. This included all available years through a series of systematic searches of the databases Medline, PubMed, SCOPUS, SPORTDiscus with Full-text and Web of Science (see Fig. 1). The databases were searched by the lead author using combinations of the key search terms: (i) "medial tibial stress syndrome" OR "MTSS" OR "shin splints" OR "exertional medial tibial pain"; (ii) "musc*", "calf” "lower*" and "leg" (see Additional file 2: Appendix 2-*search strategy*). The terms "musc*" and "lower*" were chosen to include as many articles as possible in which the authors investigated muscle, muscular or musculoskeletal structure or function and leg, lower limb and lower extremity, respectively.

### Inclusion and Exclusion Criteria

In this systematic literature review, we defined MTSS according to the criteria described by Yates et al. [6]. However, studies that used alternate terminology to define MTSS were also included in the review if the study authors had excluded participants with a tibial stress fracture, chronic exertional compartment syndrome, lower leg tendinopathy and neurovascular pathologies.

Eligibility criteria were established before beginning the search. Articles were included if they were written in English and investigated lower limb muscle structural (excluding anatomical location) or functional variables associated with MTSS injury (e.g. a measure of leg circumference or ankle plantar flexor endurance). Additional relevant published papers were then obtained from the reference lists of the sources located in the databases to help explain and support the information presented throughout this review. Articles were excluded if the authors had investigated chronic exertional compartment syndrome, tibial stress fracture, lower leg tendinopathy or shin pain of neurovascular origin.

Numerous authors have assessed ankle joint plantar flexion and dorsiflexion range of motion associated with MTSS injury [6, 26–32]. Although leg muscle structure and function can influence ankle joint range of motion, numerous other factors are also involved, such as the geometry of the articulating surfaces, the joint capsule, age and sex [33]. As this paper focussed on leg muscle structure and function, ankle joint range of motion was not included as an outcome variable.

### Procedures

The lead author assessed the methodological quality of the studies described in the articles included in the review using the modified Downs and Black Quality Assessment Checklist [34]. The Downs and Black Quality Assessment Checklist exhibits high test–retest and inter-rater reliability (*r* = 0.88 & 0.75) and criterion validity when compared with the global scores obtained using the Standards of Reporting Trials Group (*r* = 0.90) [34]. Therefore, the Downs and Black Quality Assessment Checklist is an appropriate tool to assess the methodological quality of both randomised and non-randomised health care intervention studies. The Quality Assessment Checklist has recently been modified to allow a fairer appraisal of intervention and non-intervention studies [35, 36]. The Quality Assessment Checklist reports on the bias of study reporting, internal and external validity and power. The following amendments to the Downs and Black Quality Assessment Checklist [34], modelled on the previous work of Hebert-Losier et al. [35], were implemented for this review (see Additional file 3: Appendix 3-*Modified Downs and Black Quality Assessment Checklist*). The terms 'patient' or 'subject' were replaced with 'participant' and 'treatment' interpreted in the context of testing. An additional option of 'Not Applicable' was added to several questions (4, 8–9, 12–14, 17, 19, 21–24, 26), which were deemed inappropriate to answer (i.e. the study was not an intervention study), and these questions were excluded from the total applicable points when this option was selected. Question 27 was simplified to cover statistical significance, whereby if a study reached statistical significance, it was answered 'Yes' (1 point), and if it did not reach significance, it was answered 'No' (0 points). In Question 20, an article was scored 'Yes' if its author reported or referenced the level of accuracy of the instruments used in the study. When referencing confounders in Questions 5 and 25, age, sex, athletic activity, competitive level or measure of weekly training and BMI were considered confounding variables. When assessing prospective studies, a history of MTSS was also considered a confounding variable. A score of 2 points was given if all principle confounders were reported. One point was awarded if three confounders were reported, and a score of zero was given when only two confounders were stated. All scores were then expressed, using Eq. (1), as a percentage of the total applicable points.

1 $$\frac{\mathrm{Total\,number\,of\,points}}{\mathrm{Total\,applicable\,points}} \times 100$$

Two other authors reviewed controversial articles to reach a consensus before being included in the review. Once included in the review, data concerning the study design, aims, population (e.g. military or recreational athlete), muscle structural or functional variables, or interventions were assessed along with the implications for MTSS injury. Collated data were stored in a custom Microsoft Excel® 2016 spreadsheet (Microsoft Corporation, Redmond, WA, USA).

### Statistical Analysis

Where relevant, data were pooled, and descriptive statistics were expressed as the pooled mean ± standard deviation (see Eqs. 2 and 3) to summarise available data to assist in understanding the structural and functional characteristics of the leg muscles associated with MTSS. Additional data were sought from one author who had not responded to the request at the time of publication.

2 $${\mathrm{Pooled}} {\bar{x}} =\frac{\left(\left({ {\bar{x}} }_{1}\times {n}_{1}\right)+\left({ {\bar{x}} }_{2}\times {n}_{2}\right)+\cdots \left({ {\bar{x}} }_{x}\times {n}_{x}\right)\right)}{ \Sigma n}$$

3 $$\mathrm{Pooled} \sigma =\frac{\left[\left({n}_{1}-1\right)\times {\sigma }_{1}\right]+\left[\left({n}_{2}-1\right)\times {\sigma }_{2}\right]+\cdots \left[\left({n}_{x}-1\right)\times {\sigma }_{x}\right]}{ \Sigma n-1x}$$

## Results

The initial search results from five databases are shown in Fig. 1. After applying the inclusion criteria, 3705 studies remained. After the exclusion criteria were applied, 17 studies remained and were included in this review. Of the 17 articles, there were 13 case–control studies, 3 prospective cohort studies and 1 case series. Using the modified Downs and Black Quality Assessment Checklist (see Table 1), the average score was 71.7 ± 16.4% (range 35–100%). The most common areas of poor performance with respect to the checklist were related to participants not being representative of the source population and a lack of blinding to those measuring the main outcomes variables.

**Table 1:** Modified Black and Downs Quality Assessment scores for 17 articles included in the systematic review

Article	Reporting	External validity	Bias	Confounding	Power	Score	Total	Rating (%)	Ranking
Akiyama et al. [13]	7	1	2	0	1	11	18	61.1	13
Burne et al. [10]	8	1	4	3	1	17	20	85	2
Clement et al. [14]	3	1	1	2	0	7	20	35	17
Ercan et al. [23]	7	1	2	2	0	12	17	70.6	9
Franettovich et al. [15]	8	1	3	0	1	13	18	72.2	8
Garth et al. [16]	4	0	2	0	1	7	17	41.2	16
Hubbard et al. [17]	8	1	3	4	0	16	19	84.2	3
Madeley et al. [12]	7	1	3	0	1	12	16	75	6
Moen et al. [18]	8	1	3	2	0	14	19	73.7	7
Naderi et al. [37]	7	0	4	4	1	16	20	80	4
Rathleff et al. [19]	5	1	3	1	1	11	18	61.1	13
Sabeti et al. [24]	5	1	2	1	0	9	17	52.9	15
Saeki et al. [22]	6	1	3	1	1	12	17	70.6	9
Saeki et al. [21]	6	1	3	1	1	12	17	70.6	9
Sobhani et al. [20]	7	1	3	3	0	14	18	77.8	5
Winters et al. [38]	8	1	4	3	1	17	17	100	1
Yüksel et al. [11]	7	1	3	0	1	12	17	70.6	9

Within this review, nine studies included participants from athletic populations of varying competitive levels, three studies included participants from military populations and five studies failed to report the study population, totalling 332 individuals with MTSS symptoms and 694 control participants (see Table 2). The studies included in this review were predominately case–control in design. Therefore, the authors could not establish whether the characteristics associated with leg muscle structure or function were a cause or effect of MTSS. In the three prospective studies included in this review [10, 17, 37], the structural and functional characteristics of leg muscles were assessed and prospectively followed in asymptomatic military and university athlete populations to determine whether these characteristics had implications for MTSS development. In total, 11 articles included assessments of functional characteristics [11–13, 15–17, 19, 21–23, 37] and 6 articles included assessments of structural characteristics [10, 14, 18, 20, 24, 38] of the leg muscles (see Table 3). The assessed structural characteristics were lean and maximal leg girth and tendon structure [10, 14, 18, 20, 24, 38]. Pooled mean data from three case–control studies in which maximal leg girth was assessed [14, 18, 20] revealed no significant difference between MTSS symptomatic (*n* = 51; 379.2 ± 27.8 mm) and asymptomatic individuals (*n* = 177; 378.7 ± 31.4 mm). The functional characteristics that were assessed were leg strength [11, 17, 21, 23], isotonic ankle plantar flexor endurance [12], leg muscle shear modulus [13, 22] and neuromuscular control of leg muscles [15, 16, 19, 37]. The primary variables found to be associated with MTSS included decreased lean leg girth and higher peak soleus muscle activity during propulsion and were deemed most likely to be associated with MTSS development based on the findings of the prospective studies. From case–control studies, decreased ankle plantar flexor endurance, greater isokinetic concentric eversion strength, increased leg muscle shear modulus and altered neuromuscular recruitment strategies were deemed possible risk factors. However, a causal relationship between the outcome variables identified from case–control studies and MTSS development cannot be determined (see Table 4).

**Table 2:** Characteristics of the participants tested in each of the 17 articles included in the systematic review

Article	Sample size injured v control (*n*)	Sex (*n*)	Population	Age (years)	BMI (kg/m^2^)
Akiyama et al. [13]	Inj (24), Con (20)	Male (44)	Various sports	Inj (21.9 ± 6.4), Con (19.4 ± 2.9)	Inj (23.9), Con (23.1) ^a^
Burne et al. [10]	Inj (23), Con (135)	Male (122), Female (36)	Military	Inj (18.4, 17.1 – 20.8), Con (18.4, 17.1 – 21.8)	Inj (22.4, 19.4 – 26.3), Con (21.3, 17.8 – 27.8)
Clement et al. [14]	Inj (20)	Male (12), Female (8)	Various sports	Inj (17.5, 13 – 24)	NR
Ercan et al. [23]	Inj (21), Con (12)	Male (24), Female (9)	Track and field and football	Inj (17.0 ± 0.3), Con (15.0 ± 0.3)	Inj (19.8 ± 2.3), Con (18.7 ± 1.6)
Franettovich et al. [15]	Inj (14), Con (14)	Female (28)	NR	Inj (25.9 ± 5.5), Con (25.5 ± 6.2)	Inj (22.6), Con (23.0) ^a^
Garth et al. [16]	Inj (17), Con (17)	Male (24), Female (10)	Various sports	Cohort (15.0 – 35.0)	NR
Hubbard et al. [17]	Inj (29), Con (117)	Male (65), Female (81)	Various sports	Inj (19.0 ± 0.98), Con (19.9 ± 1.8)	Inj (21.5), Con (21.4) ^a^
Madeley et al. [12]	Inj (30), Con (30)	Male (32), Female (28)	Various sports	Inj (24.0 ± 5.7), Con (22.8 ± 5.2)	Inj (23.5 ± 2.7), Con (22.8 ± 2.3)
Moen et al. [18]	Inj (15), Con (20)	Male (35)	Military	Inj (19.0 ± 1.5), Con (19 ± 1.5)	Inj (23.8 ± 2.0), Con (22.5 ± 1.6)
Naderi et al. [37]	Inj (23), Con (112)	Male (51), Female (61)	Various sports	Inj (23.1 ± 2.2), Con (22.9 ± 2.1)	Inj (21.5 ± 2.3), Con (24.0 ± 3.0)
Rathleff et al. [19]	Inj (14), Con (11)	NR	NR	Inj (27.8 ± 8.8), Con (27.3 ± 6.2)	Inj (25.8 ± 5.1), Con (24.9 ± 3.1)
Sabeti et al. [24]	Inj (17), Con (18)	NR	NR	Inj (21.1 ± 2.3), Con (20.7 ± 2.5)	Inj (21.7 ± 2.7), Con (20.7 ± 2.2)
Saeki et al. [22]	Inj (14), Con (10)	Male (24)	NR	Cohort (20.0 ± 1.7)	Cohort (19.4) ^a^
Saeki et al. [21]	Inj (15), Con (12)	Male (27)	Collegiate runners	Cohort (20.0 ± 1.6)	Cohort (19.4) ^a^
Sobhani et al. [20]	Inj (30), Con (151)	Male (181)	Military	Inj (29.5 ± 3.8), Con (30.3 ± 4.8)	Inj (26.8), Con (27.4) ^a^
Winters et al. [38]	Inj (15), Con (27)	Male (8), Female (34)	Dance athletes	Inj (20.3 ± 2.4), Con (21.1 ± 3.4)	Inj (22.3 ± 2.5), Con (21.1 ± 2.5)
Yüksel et al. [11]	Inj (11), Con (11)	Male (14), Female (8)	NR	Inj (21.0 ± 1.9), Con (23.2 ± 2.9)	Inj (22.4 ± 2.6), Con (22.0 ± 3.1)

Con, Control; Inj, Injured; NR, Not reported

^a^Author calculated from available data

**Table 3:** Summary of the study design, inclusion criteria, measured variables and main outcomes of the 17 articles included in the systematic review

Article	Study design	Injury criteria	Variables of interest	Main outcomes
Akiyama et al. [13]	Case–control	Yates et al. [6]	Lower leg muscle shear modulus	Shear Moduli of MG, LG, SOL, PL and TA was significantly greater (*p* = 0.01) in participants with MTSS compared to control group
Burne et al. [10]	Prospective cohort	Atraumatic, 7-day history of at least 10 cm of diffuse medial tibial pain	Lean lower leg girth	Men with EMTP had a significantly reduced right lean calf girth of 4.2% (*p* = 0.044) compared to control group
Clement et al. [14]	Case series	Early stage bone stress continuum with end stage being TSF	Maximal lower leg girth	Atrophy of 1.46 cm at level of maximal muscle mass of the anterior tibial group and gastrocnemius on the affected side compared to unaffected side
Ercan et al. [23]	Case–control	Atraumatic activity related diffuse (> 5 cm) pain at the distal 2/3^rd^ of the tibia	Lower leg peak isokinetic force	MTSS and control participants displayed no significant difference in isokinetic ankle plantar flexion and dorsiflexion strength
Franettovich et al. [15]	Case–control	History of ERLP within previous 12 months. Excluded if diagnosis of CECS or TSF	Lower leg muscle recruitment parameters	Individuals with a history of ERLP demonstrated a significant lower peak LG activity during stance 20.5% (*p* = 0.04) and swing 1.7% (*p* = 0.03) of MVC respectively compared to control participants
Garth et al. [16]	Case–control	Diffuse incapacitating pain at the posteromedial middle one-third of the tibia aggravated by repetitive weight bearing	MTPJ ROM	Injured participants displayed a significant increase (*p* ≤ 0.01) in sagittal plane ROM of the MTPJ, pain on flexion of FDL and mild claw toe deformity of digits 2–5
Hubbard et al. [17]	Prospective cohort	Yates et al. [6]	Lower leg muscle MVIC	MTSS and control participants demonstrated no significant difference in lower leg muscle strength measures compared to control participants
Madeley et al. [12]	Case–control	Yates et al. [6]	Standing heel raise endurance	Injured participants completed significantly less heel raise repetitions (*p* < 0.001) compared to non-injured group
Moen et al. [18]	Case–control	Yates et al. [6]	Maximal and lean lower leg girth	No significant difference in maximal or lean lower girth between injured and control participants
Naderi et al. [37]	Prospective cohort	Yates et al. [6]	Lower leg muscle recruitment parameters	Injured group displayed significantly larger peak SOL EMG amplitude during propulsion (*p* = 0.01) compared to control group
Rathleff et al. [19]	Case–control	Pain in the distal 2/3^rd^ of the posterior-medial tibia, exacerbated with repetitive weight-bearing activity	Lower leg muscle recruitment parameters	Injured group displayed significantly increased complexity of EMG signal of the TA (*p* = 0.02) and SOL (*p* = 0.01) compared to control group
Sabeti et al. [24]	Case–control	Yates et al. [6]	Maximal lower leg girth	No statistically significant difference between injured and control group
Saeki et al. [22]	Case–control	Yates et al. [6]	Lower leg muscle shear modulus	Individuals with a history of MTSS have a statistically significant higher shear modulus of FDL (*p* < 0.01) and TP (*p* < 0.05) compared to the control group
Saeki et al. [21]	Case–control	Yates et al. [6]	Lower leg muscle MVIC	Runners with a history of MTSS displayed a statistically significant higher plantarflexion MVIC torque of the 1st MTPJ (*p* = 0.04) compared to control participants
Sobhani et al. [20]	Case–control	Yates et al. [6]	Maximal lower leg girth	No statistically significant difference in maximal lower leg girth between injured and control group
Winters et al. [38]	Case–control	Yates et al. [6]	Musculoskeletal ultrasound	Tendinous abnormalities were commonly found in MTSS and asymptomatic individuals
Yüksel et al. [11]	Case–control	Yates et al. [6]	Lower leg muscle peak isokinetic force	Average eversion concentric strength was significantly higher (*p* < 0.05) in the injured group with those individuals displaying a strength imbalance whereby the evertor muscles were stronger than the invertor muscles

CECS, Chronic exertional compartment syndrome; EMG, Electromyography; EMTP, Exertional medial tibial pain; ERLP, Exercise-related leg pain; FDL, Flexor digitorum longus; LG, Lateral Gastrocnemius; MG, Medial gastrocnemius; MTPJ, Metatarsophalangeal joint; MVC, Maximal voluntary contraction; MVIC, Maximal voluntary isometric contraction; PL, Peroneus longus; ROM, Range of Motion; SOL, Soleus; TA, Tibialis anterior; TP, Tibialis posterior; TSF, Tibial stress fracture; TSS, Tibial stress syndrome

**Table 4:** Likelihood of risk factors associated with MTSS for 17 articles included in the systematic review

Likely	Possible	Not associated
Decreased lean leg girth [10]	Deficit in ankle plantar flexor endurance [12]	Leg tendon abnormality [38]
Higher peak SOL muscle activity during propulsion [37]	Greater isokinetic concentric eversion strength [11]	MVIC strength of leg muscle groups [17, 21]
	Increased shear modulus of leg muscles [13, 22]	
	Altered neuromuscular recruitment strategies [15, 19]	

MTSS, Medial tibial stress syndrome; MVIC, Maximal voluntary isometric contraction; SOL, Soleus

## Discussion

To our knowledge, this is the first systematic review to critically appraise the scientific literature pertaining to structural and functional leg muscle characteristics associated with MTSS. Despite numerous authors assessing risk factors associated with MTSS development, there is a lack of high-quality evidence to determine whether individuals with MTSS display differences in leg muscle structure or function compared to active asymptomatic individuals. Therefore, only 17 articles were deemed suitable to include within this systematic review. The primary variables found to be associated with MTSS development in the 17 studies are discussed below.

### Structural Characteristics

#### Leg Girth

Leg girth has been assessed as a risk factor for developing MTSS because it is hypothesised that the amount of leg muscle bulk will influence the ability of the leg to attenuate ground reaction forces generated at foot–ground contact [10]. Researchers have used two protocols to measure leg girth: (i) lean leg girth and (ii) maximal leg girth. Lean leg girth is the maximal leg girth measured while a participant is standing, corrected for adipose tissue thickness [10, 18]. Maximal leg girth is a standing non-corrected measure of leg girth [14, 18, 20].

In the only published prospective study identified in this field, the study authors measured the maximal leg girth of 158 military recruits (122 men and 36 women). They found a statistically significant (*p* = 0.044) 4.2% reduction in the right-sided lean leg girth of the 12 men who developed exertional medial tibial pain compared to the male military recruits who did not develop the injury (control group) [10]. The diagnostic criteria for exertional medial tibial pain included an atraumatic 7-day history of at least 10 cm of diffuse medial tibial pain. Although this definition differs from the current best practice injury definition [6], the diagnostic criteria fulfilled the key characteristics of the current best practice MTSS injury definition and was therefore included in this review. Furthermore, although not statistically significant, men who developed exertional medial tibial pain had a mean left lean leg girth that was 2.9% less compared to the control group. Although there was no significant difference between the lean leg girth of women who developed exertional medial tibial pain (*n* = 11) compared to control participants (*n* = 25), the small number of women participants reduced the statistical power of the study. In a case–control study of 15 MTSS symptomatic and 20 control male military recruits [18], the authors did not find any significant difference between the two participant groups for lean leg girth.

Pooled mean data for maximal leg girth revealed no significant difference between MTSS symptomatic and asymptomatic individuals. However, these pooled mean data should be interpreted with caution due to limitations of the methodologies used to measure maximal leg girth [14, 18, 20]. That is, Sobhani et al. [20] measured the maximal leg girth of the 181 military recruits only to the nearest 0.5 cm, resulting in imprecise data. Furthermore, the case series data presented by Clement [14] compared unilateral symptoms of MTSS individuals to the same individuals' non-affected limbs rather than to the limbs of matched control participants. Therefore, factors such as limb dominance and neuromuscular adaptations post-injury have the potential to confound these results.

Although only one study found leg girth to be associated with developing MTSS, it was prospective in design, and it scored highest on the Quality Assessment Checklist of the studies in which leg girth was assessed [10]. The remaining studies in which leg girth was assessed were case–control in design. Therefore, the authors of these case–control studies were unable to ascertain whether the lack of difference in leg girth was an effect of the injury or related to rest and subsequent morphological changes to the leg muscles. It must also be acknowledged that circumferential measures of leg girth do not compensate for variances in bone volume and adipose tissue. Therefore, future research is warranted to quantify the relative proportion of lower limb muscle, bone and adipose tissue to determine how lean leg girth affects the composition of leg muscles in vivo to help inform prevention strategies.

#### Leg Tendon Abnormalities

In combination with leg muscle composition, lower limb tendon composition plays a pivotal role in attenuating ground reaction forces at foot–ground contact and transferring forces between leg muscles and bony structures to which they attach. In vivo assessment of the deep ankle plantar flexor tendons using musculoskeletal ultrasound of MTSS symptomatic and asymptomatic dancers has been provided by Winters et al. [38]. The authors assessed the flexor hallucis longus (FHL), flexor digitorum longus (FDL) and tibialis posterior (TP) of 15 MTSS symptomatic and 27 asymptomatic matched controls for pathological changes such as the presence of intratendinous and tendon sheath hypoechoic areas or hypoechoic oedema distending from the tendon sheath [38]. The authors reported that MTSS was not a function of tendon abnormality because both MTSS symptomatic and asymptomatic dancers displayed pathological changes within the tibialis posterior tendon [38].

### Functional Characteristics

#### Leg Muscle Strength and Endurance

Assessing leg structural characteristics provides surrogate measures upon which functional characteristics of the lower limb are inferred because they do not consider the functional capacity of the leg. To address this, several research teams [11–13, 15, 17, 19, 21–23] have assessed leg muscle strength and endurance, shear modulus and neuromuscular control metrics to better understand how leg muscle function differs between individuals with MTSS symptoms and asymptomatic individuals.

Researchers have investigated the association between MTSS and leg muscle strength and endurance to provide evidence for the two main theories associated with MTSS development: (i) muscular traction inducing periostitis [39–42] and (ii) a bone stress reaction of the tibial cortex associated with repetitive tibial loading and subsequent bending resulting in posteromedial tibial bony overload [5, 43–46]. To test these theories, researchers have measured the strength of the muscles that most commonly attach to MTSS symptomatic locations and to quantify the relative strength contribution of the leg muscles to determine their ability to modulate tibial loading. This review includes one case–control study in which isotonic ankle plantar flexor endurance was measured [12] and four case–control studies in which leg muscle force was measured using dynamometry [11, 17, 21, 23]. From the results of these studies, authors have made inferences as to the potential role of these muscles in the development of MTSS.

Clinical rehabilitation protocols often incorporate ankle plantar flexion strengthening exercises into their treatment protocols [47]. The rationale for improving ankle plantar flexion endurance in MTSS symptomatic individuals is provided by Madeley et al. [12] who assessed 30 MTSS symptomatic individuals (median symptom duration 15 weeks) and 30 sex, age and BMI matched controls. The authors reported that MTSS symptomatic individuals were able to complete significantly fewer single-leg heel raises (23 ± 5.6) compared to control participants (33 ± 8.6; *p* ≤ 0.001). Due to the potential influence of pain on the number of heel raises completed, future prospective studies are required to determine whether the reduced ankle plantar flexion endurance capacity of MTSS symptomatic individuals was a cause or effect of MTSS.

Several authors have also assessed the strength of leg muscles of individuals with MTSS using dynamometry. The studies by Hubbard et al. [17] and Saeki et al. [21] demonstrated that individuals who developed and had a history of MTSS displayed no statistically significant difference in the maximal voluntary isometric contraction (MVIC) strength of the leg plantar flexor, dorsiflexor, invertor or evertor muscle groups when compared to control individuals. Individuals with a history of MTSS, however, displayed a significantly greater MVIC plantar flexion torque of the FHL (12.0 ± 3.0 Nm) compared to asymptomatic controls (9.8 ± 2.3 Nm; *p* = 0.04) [21]. However, Saeki et al. [21] concluded that the FHL is not likely to be related to the development of MTSS because the FHL does not connect to the tibial fascia. Individuals with MTSS have also been shown to display, on average, a significantly greater isokinetic concentric eversion strength (*p* < 0.05) [11], although there was no difference in isokinetic dorsiflexion and plantar flexion strength compared to asymptomatic individuals [23]. Based on these results, the authors of the study concluded that MTSS symptomatic individuals had a strength imbalance between the invertor and evertor muscles, whereby the evertor muscles were stronger [11]. These findings should be interpreted with caution, however, because age of activity initiation (*p* < 0.056) and training volume (*p* < 0.001) were significantly different between the two participant groups, with the control group beginning activity at an earlier age and having a greater training volume than their counterparts with MTSS.

Current evidence suggests that individuals with MTSS have a reduced isotonic ankle plantar flexor endurance capacity compared to asymptomatic matched controls. However, these findings are not supported by a reduced strength of the ankle plantar flexor muscle group when assessed using dynamometry. Assessing the lower limb plantar flexor, dorsiflexor, invertor and evertor muscle groups as a functional unit could mask variability in the strength of individual muscles within that functional unit in MTSS symptomatic compared to asymptomatic controls. Potentially, individuals who are susceptible to MTSS could employ compensatory muscle recruitment strategies to produce comparable force to those who do not develop MTSS. Therefore, future prospective research is warranted to assess ankle plantar flexor endurance and individual leg muscle strength to elucidate whether specific ankle plantar flexor muscles are responsible for the reduced isotonic ankle plantar flexor endurance capacity seen in individuals with MTSS. Furthermore, although current research indicates that a strength imbalance between the invertor and evertor muscles is apparent during isokinetic but not isometric contraction in individuals with MTSS, whether this is a cause or effect of MTSS requires further prospective assessment.

#### Leg Muscle Shear Modulus

Although reduced ankle dorsiflexion range of motion is not reported to be a risk factor associated with MTSS development [8, 9], MTSS symptomatic individuals commonly report a sensation of increased muscle tightness of the ankle joint plantar flexors [8]. Two case–control studies [13, 22] were reviewed in which the research teams used shear wave elastography to quantify leg muscle tightness in men, represented via the shear modulus of the muscles of the superficial posterior compartment of the leg (lateral gastrocnemius (LG), medial gastrocnemius (MG), soleus (SOL) and peroneus longus (PL)). Akiyama et al. [13] also assessed an antagonist to ankle plantar flexion, the tibialis anterior (TA), whereas Saeki et al. [22] specifically assessed the muscles contributing to ankle plantar flexion, and therefore also included FDL, FHL, peroneus brevis (PB) and TP. Although both studies identified an increase in the shear modulus of several muscles in symptomatic participants relative to controls, there was a lack of consistency in the muscles identified between the studies. Akiyama et al. [13] concluded that individuals with MTSS had a greater shear modulus of the LG, MG, PL, SOL and TA, whereas Saeki et al. [22] concluded that only the FDL and TP demonstrated an increase in shear modulus in individuals with MTSS compared to asymptomatic controls. These between-study differences in results can be attributed to several factors, including different participant inclusion criteria. For example, Akiyama et al. [13] assessed individuals with MTSS who were symptomatic at the time of testing, whereas, in order to avoid the confounding variable of pain, Saeki et al. [22] assessed individuals with a history of MTSS, excluding individuals with pain at the time of testing. Both research teams also used different metrics to report the shear modulus, preventing the pooling of the data. Despite these limitations, conclusions drawn from the available data suggest an increase in shear modulus of the lower limb muscles, particularly of the ankle plantar flexors, in MTSS symptomatic men that persists following recovery of symptoms. For more meaningful conclusions to be reached, future prospective research, including both men and women, is required to avoid the confounding variable of pain on shear modulus and determine whether a causal relationship can be established between the shear modulus of the leg muscles and the development of MTSS.

#### Neuromuscular Control

Much of the previous research examining functional characteristics of the leg that might predispose individuals to MTSS has been limited by the experimental task being non-weight bearing or not activity specific for the individual (e.g. isometric or isokinetic muscle contraction) [11, 13, 17, 21–23]. One method to better understand the functional capacity of the leg muscles is to monitor the neuromuscular control of these muscles during dynamic tasks, such as running or walking. One prospective and two case–control studies were located in which neuromuscular control of the leg muscles in individuals with MTSS and asymptomatic individuals were assessed [15, 19, 37]. In a prospective study, Naderi et al. [37] assessed SOL and TA electromyographic (EMG) signals during the stance phase of running of 112 active university students. During a 17-week follow-up period, 23 (9 men and 14 women) individuals were diagnosed with MTSS. The authors found a statistically significant higher peak SOL EMG amplitude during absorption (*p* = 0.01) and propulsion (*p* = 0.02) in the MTSS group compared to the control group, although no significant difference was found for TA. Using a stepwise logistic regression analysis, the authors concluded that higher peak EMG amplitude of the SOL during propulsion significantly increased the risk of MTSS development by 5% (*p* = 0.01). The authors hypothesised that the increased SOL EMG amplitude during propulsion could induce greater SOL traction on the posteromedial tibia, particularly in individuals with a higher dynamic foot posture due to greater and prolonged SOL contraction [37]. In a case–control comparison, Franettovich et al. [15] assessed EMG signals of 12 lower limb muscles and lower limb kinematics while 14 female participants who had a history of exercise-related leg pain within the previous 12 months and 14 sex, age, height and weight-matched asymptomatic controls walked on a treadmill. Individuals with a history of exercise-related leg pain demonstrated a significant (*p* = 0.048) reduction in LG peak activity by 20.5% and 1.7% MVIC during stance and swing. Despite differences in peak LG muscle activity, the authors observed no differences in foot posture, foot mobility or motion at the pelvis, hip, knee and ankle between the participant groups. Franettovich et al. [15] hypothesised that lower peak LG activity could potentially increase posteromedial tibial loading and MTSS development or be a compensatory effect of exercise related leg pain symptoms. In addition, Rathleff et al. [19] assessed SOL and TA EMG signals along with midfoot kinematics from 14 MTSS symptomatic and 11 asymptomatic controls during treadmill walking. Rathleff et al. [19] reported that MTSS symptomatic individuals displayed a significant increase in complexity of the TA (*p* = 0.01) and SOL (*p* = 0.02) EMG signals with a lower complexity of midfoot kinematics compared to control participants. Complexity was defined as the degree of variability within the signal, where lower values indicated a more regular signal and higher values indicated an increase in randomness in the signal [19]. Rathleff et al. [19] concluded that the increased complexity of the TA and SOL EMG signals and lower midfoot kinematic complexity in the MTSS symptomatic group were associated with less movement variability. Increased complexity of EMG activity is hypothesised to be caused by the effects of adaptation due to pathology [48]. Contention remains, however, as to whether the neuromuscular system up or down-regulates in response to pathology [49, 50]. This finding supports the notion of Hamill et al. [51], who hypothesised that increased tissue stress in patellofemoral pain was caused by individuals who experienced a narrow range of motion with less movement variability compared to healthy controls. Lower limb muscle EMG signal adaptations reported by Naderi et al. [37], Franettovich et al. [15] and Rathleff et al. [19] build on the earlier work of Garth et al. [16], who demonstrated that individuals with posteromedial shin pain displayed significant impairment of intrinsic muscles controlling the toe flexors and extensors compared to control participants. Furthermore, the findings by Franettovich et al. [15] and Rathleff et al. [19] are also consistent with previous research in which individuals with MTSS were able to produce force output that was comparable to asymptomatic individuals [17, 21], although possibly using compensatory muscle recruitment strategies to achieve this.

Findings of the three studies included for review demonstrate that altered leg muscle recruitment strategies of the triceps surae are associated with MTSS injury. The findings of Naderi et al. [37] build on earlier work by Franettovich et al. [15] who assessed the SOL, LG and MG and concluded that when matched to controls, MTSS symptomatic individuals displayed lower peak LG muscle activation only. Building on the findings of Naderi et al. [37], it could be hypothesised that once individuals experience MTSS pain, they alter their neuromuscular patterning to reduce SOL muscle activation and associated muscular traction on the tibia as a pain management strategy. To substantiate this notion, however, further prospective assessment of neuromuscular patterning of the triceps surae by individuals who develop MTSS is required.

#### Limitations

The primary limitation of this review is that most studies included for appraisal were case–control in design, limiting our ability to determine whether the assessed factors were a cause or effect of MTSS. In this review, we identified decreased lean leg girth as a likely risk factor associated with developing MTSS. We acknowledge, however, that decreased lean leg girth does not account for variances in tibial bone volume. Furthermore, a lack of consistent testing procedures and outcome variables in the studies we reviewed reduced our ability to pool a greater proportion of data, limiting our ability to draw firm conclusions.

## Conclusions

The key findings from the prospective studies included for review indicate that decreased lean leg girth is a likely risk factor for developing MTSS [10], although this reduction in muscle girth was not related to the capacity of the leg muscles to produce maximal force [17]. Furthermore, higher peak soleus muscle activity during propulsion is associated with an increased risk of MTSS development [37]. Cross-sectional comparison of individuals suffering MTSS compared to asymptomatic controls demonstrated deficits in ankle plantar flexor endurance, greater isokinetic concentric eversion strength, increased muscle shear modulus and altered neuromuscular recruitment strategies. However, whether these differences between symptomatic patients and controls were due to pain or associated with injury development needs to be further explored.

This review highlights large knowledge gaps in the available literature investigating how leg muscle structure and function are associated with MTSS development. These knowledge gaps are due to the lack of prospective studies, which are required to identify a causal relationship between risk factors and MTSS development, and the failure of researchers to mitigate the confounding variable of pain. Future research should assess specific structural characteristics of the leg, such as the relative proportion of in vivo lower limb muscle, bone and adipose tissue, to determine whether specific muscles are responsible for the reduction in lean leg girth between individuals who develop MTSS compared to asymptomatic controls. Finally, future research should assess ankle plantar flexor endurance, individual leg muscle strength and the neuromuscular patterning of the triceps surae during running to better inform MTSS injury prevention strategies and rehabilitation protocols.

## Supplementary Information

**Additional file 1.** Appendix 1.

**Additional file 2.** Appendix 2. DOCTYPE ( ar ) AND ( LIMIT-TO ( LANGUAGE, “English” ) )

**Additional file 3.** Appendix 3.

## Data Availability

All data generated or analysed during this study are included in this published article.

## Abbreviations

BMI: Body mass index
EMG: Electromyographic
FDL: Flexor digitorum longus
FHL: Flexor hallucis longus
LG: Lateral gastrocnemius
MG: Medial gastrocnemius
MTSS: Medial tibial stress syndrome
MVIC: Maximal voluntary isometric contraction
PB: Peroneus brevis
PL: Peroneus longus
PRISMA-P: Preferred reporting items for systematic reviews and meta-analysis protocols
SOL: Soleus
SD: Standard deviation
TA: Tibialis anterior
TP: Tibialis posterior

## References

[1] Kozlovskaia M, Vlahovich N, Rathbone E, Manzanero S, Keogh J, Hughes DC (2019). A profile of health, lifestyle and training habits of 4720 Australian recreational runners—the case for promoting running for health benefits. Health Promot J Aust.

[2] Lavie CJ, Lee D, Sui X, Arena R, O'Keefe JH, Church TS (2015). Effects of running on chronic diseases and cardiovascular and all-cause mortality. Mayo Clin Proc.

[3] Kluitenberg B, van Middelkoop M, Diercks R, van der Worp H (2015). What are the differences in injury proportions between different populations of runners? A systematic review and meta-analysis. Sports Med.

[4] Van Middelkoop M, Kolkman J, Van Ochten J, Bierma-Zeinstra SM, Koes B (2008). Prevalence and incidence of lower extremity injuries in male marathon runners. Scand J Med Sci Sports.

[5] Moen MH, Tol JL, Weir A, Steunebrink M, De Winter TC (2009). Medial tibial stress syndrome a critical review. Sports Med.

[6] Yates B, White S (2004). The incidence and risk factors in the development of medial tibial stress syndrome among naval recruits. Am J Sport Med.

[7] Bennett JE, Reinking MF, Pluemer B, Pentel A, Seaton M, Killian C (2001). Factors contributing to the development of medial tibial stress syndrome in high school runners. J Orthop Sports Phys Ther.

[8] Newman P, Witchalls J, Waddington G, Adams R (2013). Risk factors associated with medial tibial stress syndrome in runners: a systematic review and meta-analysis. Open Access J Sports Med.

[9] Winters M, Eskes M, Weir A, Moen MH, Backx FJG, Bakker EWP (2013). Treatment of medial tibial stress syndrome: a systematic review. Sports Med.

[10] Burne SG, Khan KM, Boudville PB, Mallet RJ, Newman PM, Steinman LJ (2004). Risk factors associated with exertional medial tibial pain: a 12 month prospective clinical study. Br J Sports Med.

[11] Yüksel O, Ozgurbuz C, Ergun M, Islegen C, Taskiran E, Denerel N (2011). Inversion/eversion strength dysbalance in patients with medial tibial stress syndrome. J Sport Sci Med.

[12] Madeley LT, Munteanu SE, Bonanno DR (2007). Endurance of the ankle joint plantar flexor muscles in athletes with medial tibial stress syndrome: a case-control study. J Sci Med Sport.

[13] Akiyama K, Akagi R, Hirayama K, Hirose N, Takahashi H, Fukubayshi T (2016). Shear modulus of the lower leg muscles in patients with medial tibial stress syndrome. Ultrasound Med Biol.

[14] Clement DB (1974). Tibial stress syndrome in athletes. Am J Sport Med.

[15] Franettovich M, Chapman AR, Blanch P, Vicenzino B (2010). Altered neuromuscular control in individuals with exercise-related leg pain. Med Sci Sports Exerc.

[16] Garth WP, Miller ST (1989). Evaluation of claw toe deformity, weakness of the foot intrinsics and posteromedial shin pain. Am J Sport Med.

[17] Hubbard TJ, Carpenter EM, Cordova ML (2009). Contributing factors to medial tibial stress syndrome: a prospective investigation. Med Sci Sports Exerc.

[18] Moen MH, Bongers T, Bakker EW, Zimmermann WO, Weir A, Tol JL (2012). Risk factors and prognostic indicators for medial tibial stress syndrome. Scand J Med Sci Sports.

[19] Rathleff MS, Samani A, Olesen CG, Kersting UG, Madeleine P (2011). Inverse relationship between the complexity of midfoot kinematics and muscle activation in patients with medial tibial stress syndrome. J Electromyogr Kinesiol.

[20] Sobhani V, Shakibaee A, Aghda AK, Meybodi MKE, Delavari A, Jahandideh D (2015). Studying the relation between medial tibial stress syndrome and anatomic and anthropometric characteristics of military male personnel. Asian J Sports Med.

[21] Saeki J, Nakamura M, Nakao S, Fujita K, Yanase K, Morishita K (2017). Ankle and toe muscle strength characteristics in runners with a history of medial tibial stress syndrome. J Foot Ankle Res.

[22] Saeki J, Nakamura M, Nakao S, Fujita K, Yanase K, Ichihashi N (2018). Muscle stiffness of posterior lower leg in runners with a history of medial tibial stress syndrome. Scand J Med Sci Sports.

[23] Ercan S (2019). Ankle isokinetic muscle strength and navicular drop in athletes with medial tibial stress syndrome. Cukurova Med J.

[24] Sabeti V, Khoshraftar YN, Bijeh N (2019). The relationship between shin splints with anthropometric characteristics and some indicators of body composition. J Sports Med Phys Fit.

[25] Moen MH, Holtslag L, Bakker E, Barten C, Weir A, Tol JL (2012). The treatment of medial tibial stress syndrome in athletes; a randomized clinical trial. BMC Sports Sci Med Rehabil.

[26] Andrish JT, Bergfeld JA, Walheim J (1974). A prospective study on the management of shin splints. J Bone Joint Surg Am.

[27] Bartosik KE, Sitler M, Hillstrom HJ, Palamarchuk H, Huxel K, Kim E (2010). Anatomical and biomechanical assessments of medial tibial stress syndrome. J Am Podiatr Med Assoc.

[28] Becker J, James S, Wayner R, Osternig L, Chou LS (2017). Biomechanical factors associated with achilles tendinopathy and medial tibial stress syndrome in runners. Am J Sport Med.

[29] Becker J, Nakajima M, Wu WFW (2018). Factors contributing to medial tibial stress syndrome in runners: a prospective study. Med Sci Sports Exerc.

[30] Garnock C, Witchalls J, Newman P (2018). Predicting individual risk for medial tibial stress syndrome in navy recruits. J Sci Med Sport.

[31] Loudon JK, Dolphino MR (2010). Use of foot orthoses and calf stretching for individuals with medial tibial stress syndrome. Foot Ankle Spec.

[32] Yagi S, Muneta T, Sekiya I (2013). Incidence and risk factors for medial tibial stress syndrome and tibial stress fracture in high school runners. Knee Surg Sports Traumatol Arthrosc.

[33] Grimston SK, Nigg BM, Hanley DA, Engsberg JR (1993). Differences in ankle joint complex range of motion as a function of age. Foot Ankle.

[34] Downs SH, Black N (1998). The feasibility of creating a checklist for the assessment of the methodological quality both of randomised and non-randomised studies of health care interventions. J Epidemiol Commun Health.

[35] Hebert-Losier K, Supej M, Holmberg HC (2014). Biomechanical factors influencing the performance of elite alpine ski racers. Sports Med.

[36] Forsyth JR, Riddiford-Harland DL, Whitting JW, Sheppard JM, Steele JR (2019). Essential skills for superior wave-riding performance: a systematic review. J Strength Cond Res.

[37] Naderi A, Moen MH, Degens H (2020). Is high soleus muscle activity during the stance phase of the running cycle a potential risk factor for the development of medial tibial stress syndrome? A prospective study. J Sports Sci.

[38] Winters M, Bon P, Bijvoet S, Bakker EWP, Moen MH (2017). Are ultrasonographic findings like periosteal and tendinous edema associated with medial tibial stress syndrome? A case-control study. J Sci Med Sport.

[39] Stickley CD, Hetzler RK, Kimura IF, Lozanoff S (2009). Crural fascia and muscle origins related to medial tibial stress syndrome symptom location. Med Sci Sports Exerc.

[40] Brown AA (2016). Medial tibial stress syndrome: muscles located at the site of pain. Scientifica.

[41] Beck BR, Osternig LR, Oregon E (1994). Medial tibial stress syndrome—the location of muscles in the leg in relation to symptoms. J Bone Joint Surg Am.

[42] Bouche RT, Johnson CH (2007). Medial tibial stress syndrome (tibial fasciitis)—a proposed pathomechanical model involving fascial traction. J Am Podiatr Med Assoc.

[43] Beck BR (1998). Tibial stress injuries—an aetiological review for the purposes of guiding management. Sports Med.

[44] Batt ME, Ugalde V, Anderson MW, Shelton DK (1998). A prospective controlled study of diagnostic imaging for acute shin splints. Med Sci Sports Exerc.

[45] Gaeta M, Minutoli F, Scribano E, Ascenti G, Vinci S, Bruschetta D (2005). CT and MR imaging findings in athletes with early tibial stress injuries: comparison with bone scintigraphy findings and emphasis on cortical abnormalities. Radiology.

[46] Fredericson M, Bergman AG, Hoffman KL, Dillingham MS (1995). Tibial stress reaction in runners—correlation of clinical symptoms and scintigraphy with a new magnetic resonance imaging grading system. Am J Sport Med.

[47] Elias J (2012). Case study: medial tibial stress syndrome. SportEX Med.

[48] Bartlett R, Wheat J, Robins M (2007). Is movement variability important for sports biomechanists?. Sport Biomech.

[49] Stergiou N, Harbourne R, Cavanaugh J (2006). Optimal movement variability: a new theoretical perspective for neurologic physical therapy. J Neurol Phys Ther.

[50] Jordan K, Challis JH, Newell KM (2007). Walking speed influences on gait cycle variability. Gait Posture.

[51] Hamill J, van Emmerik REA, Heiderscheit BC, Li L (1999). A dynamical systems approach to lower extremity running injuries. Clin Biomech.

