# Supplementary material for: Are Leg Muscle, Tendon and Functional Characteristics Associated with Medial Tibial Stress Syndrome? A Systematic Review
Source: Sports Med Open. 2021 Oct 9;7:71. doi: 10.1186/s40798-021-00362-2 (PMC8502183; doi:10.1186/s40798-021-00362-2)
Supplement: Supplementary file 2 — Additional file 2. Appendix 2. DOCTYPE ( ar ) AND ( LIMIT-TO ( LANGUAGE, “English” ) ) [file 40798_2021_362_MOESM2_ESM.docx]

**Appendix 2**

DOCTYPE ( ar ) AND ( LIMIT-TO ( LANGUAGE, “English” ) )

| Item number | Search terms | Number of search results |
| --- | --- | --- |
| 1 | Medial tibial stress syndrome | 1039 |
| 2 | MTSS |  |
| 3 | Shin splints |  |
| 4 | Exertional medial tibial pain |  |
| 5 | (1) OR (2) OR (3) OR (4) AND Musc* | 172 |
| 6 | (1) OR (2) OR (3) OR (4) AND Calf | 12 |
| 7 | (1) OR (2) OR (3) OR (4) AND Lower* | 240 |
| 8 | (1) OR (2) OR (3) OR (4) AND Leg | 189 |
| 9 | (5) AND Structure OR Size OR Architecture OR Characteristics | 32 |
| 10 | (6) AND Structure OR Size OR Architecture OR Characteristics | 0 |
| 11 | (7) AND Structure OR Size OR Architecture OR Characteristics | 43 |
| 12 | (8) AND Structure OR Size OR Architecture OR Characteristics | 31 |
| 13 | (5) AND Endurance OR Strength OR Electromyography OR Stretch* OR Function OR Exercise | 97 |
| 14 | (6) AND Endurance OR Strength OR Electromyography OR Stretch* OR Function OR Exercise | 9 |
| 15 | (7) AND Endurance OR Strength OR Electromyography OR Stretch* OR Function OR Exercise | 119 |
| 16 | (8) AND Endurance OR Strength OR Electromyography OR Stretch* OR Function OR Exercise | 106 |

Search strategy for Web of Science
