# Supplementary material for: Are Leg Muscle, Tendon and Functional Characteristics Associated with Medial Tibial Stress Syndrome? A Systematic Review
Source: Sports Med Open. 2021 Oct 9;7:71. doi: 10.1186/s40798-021-00362-2 (PMC8502183; doi:10.1186/s40798-021-00362-2)
Supplement: Supplementary file 3 — Additional file 3. Appendix 3. [file 40798_2021_362_MOESM3_ESM.docx]

**Appendix 3**

| Question | Category | Downs et al. [34] | Modified version |
| --- | --- | --- | --- |
| 1 | Reporting | Is the hypothesis/aim/objective of the study clearly described?  Yes = 1  No = 0 | - Unchanged |
| 2 | Reporting | Are the main outcomes to be measured clearly described in the Introduction or Methods section?  Yes = 1  No = 0 | - Unchanged |
| 3 | Reporting | Are the characteristics of the patients included in the study clearly described?  Yes = 1  No = 0 | - Modified text: “patients” replaced with “participants” |
| 4 | Reporting | Are the interventions of interest clearly described?  Yes = 1  No = 0 | - Additional scoring option of N/A added |
| 5 | Reporting | Are the distributions of principal confounders in each group of subjects to be compared clearly described?  Yes = 2  Partly = 1  No = 0 | - Modified text: “subjects” replaced with “participants” - Confounding variables: age, sex, athletic activity, competitive level, measure of weekly training, BMI. For a prospective study, a history of MTSS |
| 6 | Reporting | Are the main findings of the study clearly described?  Yes = 1  No = 0 | - Unchanged |
| 7 | Reporting | Does the study provide estimates of the random variability in the data for the main outcomes?  Yes = 1  No = 0 | - Unchanged |
| 8 | Reporting | Have all important adverse events that may be a consequence of the intervention been reported?  Yes = 1  No = 0 | - Additional scoring option of N/A added |
| 9 | Reporting | Have the characteristics of patients lost to follow-up been described?  Yes = 1  No = 0 | - Modified text: “patient” replaced with “participant” - Additional scoring option of N/A added |
| 10 | Reporting | Have actual probability values been reported (e.g. 0.035 rather than <0.05) for the main outcomes except where the probability value is less than 0.001?  Yes = 1  No = 0 | - Unchanged |
| 11 | External validity | Were the subjects asked to participate in the study representative of the entire population from which they were recruited?  Yes = 1  No = 0  Unable to determine = 0 | - Modified text: “subjects” replaced with “participants” |
| 12 | External validity | Were those subjects who were prepared to participate representative of the entire population from which they were recruited?  Yes = 1  No = 0  Unable to determine = 0 | - Modified text: “subjects” replaced with “participants” - Additional scoring option of N/A added |
| 13 | External validity | Were the staff, places, and facilities where the patients were treated, representative of the testing the majority of patients receive?  Yes = 1  No = 0  Unable to determine = 0 | - Modified text: “patients” replaced with “participant” - Treatment interpreted in context of testing - Additional scoring option of N/A added |
| 14 | Bias | Was an attempt made to blind study subjects to the intervention they have received?  Yes = 1  No = 0  Unable to determine = 0 | - Modified text: “subjects” replaced with “participants” - Additional scoring option of N/A added |
| 15 | Bias | Was an attempt made to blind those measuring the main outcomes of the intervention?  Yes = 1  No = 0  Unable to determine = 0 | - Unchanged |
| 16 | Bias | If any of the results of the study were based on “data dredging”, was this made clear?  Yes = 1  No = 0  Unable to determine = 0 | - Unchanged |
| 17 | Bias | In trials and cohort studies, do the analyses adjust for different lengths of follow-up of patients, or in case-control studies, is the time period between the intervention and outcome the same for cases and controls?  Yes = 1  No = 0  Unable to determine = 0 | - Modified text: “patients” replaced with “participants” - Additional scoring option of N/A added |
| 18 | Bias | Were the statistical tests used to assess the main outcomes appropriate?  Yes = 1  No = 0  Unable to determine = 0 | - Unchanged |
| 19 | Bias | Was compliance with the intervention/s reliable?  Yes = 1  No = 0  Unable to determine = 0 | - Additional scoring option of N/A added |
| 20 | Bias | Were the main outcome measures used accurate (valid and reliable)?  Yes = 1  No = 0  Unable to determine = 0 | - Unchanged |
| 21 | Confounding | Were the patients in different intervention groups (trials and cohort studies) or were the cases and controls (case-control studies) recruited from the same population?  Yes = 1  No = 0  Unable to determine = 0 | - Modified text: “patients” replaced with “participants” - Additional scoring option of N/A added |
| 22 | Confounding | Were study subjects in different intervention groups (trials and cohort studies) or were the cases and controls (case-control studies) recruited over the same period of time?  Yes = 1  No = 0  Unable to determine = 0 | - Modified text: “subjects” replaced with “participants” - Additional scoring option of N/A added |
| 23 | Confounding | Were study subjects randomised to intervention groups?  Yes = 1  No = 0  Unable to determine = 0 | - Modified text: “subjects” replaced with “participants” - Additional scoring option of N/A added |
| 24 | Confounding | Was the randomised intervention assignment concealed from both patients and health care staff until recruitment was complete and irrevocable?  Yes = 1  No = 0  Unable to determine = 0 | - Modified text: “patients” replaced with “participants” - Additional scoring option of N/A added |
| 25 | Confounding | Was there adequate adjustment for confounding in the analyses from which the main findings were drawn?  Yes = 1  No = 0  Unable to determine = 0 | - Confounding variables: age, sex, athletic activity, competitive level, measure of weekly training, BMI. For a prospective study, a history of MTSS |
| 26 | Confounding | Were losses of patients to follow-up taken into account?  Yes = 1  No = 0  Unable to determine = 0 | - Modified text: “patients” replaced with “participants” - Additional scoring option of N/A added |
| 27 | Power | Did the study have sufficient power to detect a clinically important effect where the probability value for a difference being due to chance is less than 5%?   \|  \| Size of smallest intervention group \|  \| \| --- \| --- \| --- \| \| A \| <n_1_ \| 0 \| \| B \| n_1_-n_2_ \| 1 \| \| C \| n_3_-n_4_ \| 2 \| \| D \| n_5_-n_6_ \| 3 \| \| E \| n_7_-n_8_ \| 4 \| \| F \| n_8_+ \| 5 \| | - Scoring modified: if a study reached statistical significance = Yes. If it did not reach significance = No. - Yes = 1 - No = 0 |

Modified Downs and Black Quality Assessment Checklist
